# Sexually transmitted diseases in Ukraine.

**DOI:** 10.3201/eid0503.990332

**Published:** 1999

**Authors:** D I Ivanov


epidemics occur (e.g., diphtheria in eastern Europe in the early and mid-1990s) (9). If needed, the World Health Organization can provide information on confirmed and unconfirmed epidemics on a weekly basis.

Travel and peacekeeping mission statistics share similarities. In Namibia, the South African Armed Forces had most often observed hepatitis (unspecified), with rare cases of tuberculosis, typhoid, and meningitis (unpub. SAMS report: Disease Profile of South West Africa, 1989), as did the UN mission to Namibia, where within 12 months and with 7,114 employees, seven cases of hepatitis (mostly hepatitis A, some unspecified) occurred (10). No other vaccine-preventable infections were diagnosed in this UN mission.

Considering both risk (on the basis of incidence rates) and impact of infection, the priority for immunization (from highest to lowest) is as follows: hepatitis A, hepatitis B, rabies, poliomyelitis, yellow fever, typhoid fever, influenza, diphtheria, tetanus, meningococcal disease, Japanese encephalitis, cholera, and measles. To administer all vaccines would be extremely costly and may also result in an increased rate of adverse side-effects. Immunizations against the more frequent, more severe infections should be given priority.

If a mission is limited to one season, environmental factors of that respective season should be considered. This general rule is more important for vector-borne than for vaccine-preventable infections, except for influenza and meningococcal disease.

Persons who are already immune (because of previous immunization or immunity after infection) need not be vaccinated. The latter cause is particularly often true of hepatitis A; troops recruited in developing countries have an anti-hepatitis A virus seroprevalence rate close to 100% (11). Hepatitis B immunization, except for non- and low-responders, probably grants lifelong protection (12); the same is likely for measles vaccine.

Sometimes the host country may require proof of some specific vaccination based on the International Health Regulations (13), currently under fundamental revision to become a more effective tool in preventing the spread of infections that may be a global hazard (14).

In addition to adequate epidemiologic information and coordination between the military, international health organizations, and the host country, successful intervention efforts require thorough knowledge of vaccine characteristics with varying rates of efficacy and duration of protection. Cost-benefit evaluations, which would be very desirable, are unlikely in areas of political instability.

**Robert Steffen**
Institute for Social and Preventive Medicine of the University, Zurich, Switzerland

### References

1. DuPont HL, Ericsson C. Prevention and treatment of travelers' diarrhea. Drug Therapy 1993;328:1821-7.
2. Farthing MJG, DuPont HL, Guandalini S, Keusch GT, Steffen R. Treatment and prevention of travellers' diarrhoea. Gastroenterology International 1992;5:162-75.
3. Levine MM, Svennerholm A-M. Prioritization of vaccines to prevent enteric infections. In: DuPont HL, Steffen R, editors. Textbook of travel medicine. 1st ed. Hamilton: B.C. Becker Inc.; 1997. p. 370.
4. Steffen R, Kane MA, Shapiro CN, Schoellhorn JK, Van Damme P. Epidemiology and prevention of hepatitis A in travelers. JAMA 1994;272:885-9.
5. World Health Organization. International travel and health. Geneva: The Organization; 1999.
6. Steffen R. Risk of hepatitis B for travellers. Vaccine 1990;8:31-2.
7. Hatz CF, Bidaux JM, Eichenberger K, Mikulics U, Junghanss T. Circumstances and management of 72 animal bites among long-term residents in the tropics. Vaccine 1994;13:811-5.
8. Steffen R. Travel medicine prevention based on epidemiological data. Trans R Soc Trop Med Hyg 1991;85:156-62.
9. Hardy IRB, Dittmann S, Sutter RW. Current situation and control strategies for resurgence of diphtheria in newly independent states of the former Soviet Union. Lancet 1996;347:1739-44.
10. Steffen R, Desaules M, Nagel J, Vuillet F, Schubarth P, Jeanmaire C-H, et al. Epidemiological experience in the mission of the United Nations Transition Assistance Group (UNTAG) in Namibia. Bull World Health Organ 1992;70:129-33.
11. Centers for Disease Control and Prevention. Hepatitis A immunization. MMWR Morb Mortal Wkly Rep 1996;45(RR-15):7.
12. Hall AJ. Hepatitis B vaccination: protection for how long and against what. BMJ 1993;307:276-7.
13. World Health Organization. International health regulations. 3rd annotated ed. Geneva: The Organization; 1983.
14. World Health Organization. Revision of the international health regulations. Wkly Epidemiol Rec 1997;72:213-5.

### Sexually Transmitted Diseases in Ukraine

**To the Editor:** With the political changes in eastern Europe in the last 10 years have come social and economic changes (1). Ukraine not only faces almost insurmountable problems as it tries to form a new government, it also faces many serious health issues including sexually transmitted diseases (STDs).

Surveillance data from the Ukrainian STD Center from January 1, 1989, through December 31, 1995, were analyzed on the basis of reports received through 1997. In western Europe, the incidence of syphilis and gonorrhea declined from 1980 to 1991 to less than 2% per 100,000 persons for syphilis and less than 20% per 100,000 persons for gonorrhea. However, in Ukraine, since 1989, the notification rate of syphilis has skyrocketed—from 5 per 100,000 persons in 1990 to 170 in 1995. In some regions, this rate exceeds 220 cases per 100,000 persons. Moreover, cases among children younger than 14 years of age are also increasing. In 1995, the syphilis rate for persons older than 30 years of age was 170 per 100,000; 600 per 100,000 girls younger than 15 years of age; and 1,550 to 2,000 per 100,000 girls 15 to 16 years of age. The large number of girls with the disease is in part due to teenage prostitution (1).

Most syphilis and gonorrhea cases are attributed to sexual transmission. Explanations of this phenomenon include the rapid growth of the sex industry, increasing numbers of homeless persons and refugees in Ukrainian cities, poor diagnostic facilities, punitive legislation that reduces the likelihood of going to treatment services, and limited or inadequate treatment (2).

The Ukrainian government is reviewing its arrangements for the control of STDs, including HIV/AIDS, to identify clear objectives and priorities. Education and treatment would be effective in preventing the spread of STDs in Ukraine, but these measures are inadequately funded (3). Evaluation and risk reduction are also great weapons in preventing the spread of STDs (4). However, the response of the local and world communities has been inadequate in stemming a major STD epidemic in Ukraine.

United Nation's Children's Fund (UNICEF) is developing a long-term program in Ukraine with a focus on STDs in adolescents and youth. This comprehensive program will tackle not only STDs but other related issues, such as HIV and teenagers' reproductive health (5).

Greater coordination of the agencies responsible for STD control in Ukraine will be sought, together with an expansion of health promotion and prevention projects for young persons and groups at high risk (6). An effective strategy for the control of STDs in Ukraine will, therefore, need to find ways to modify current programs and the way they interact to create effective control interventions.

**Dmitry I. Ivanov**
University of Alabama at Birmingham, Birmingham, Alabama, USA

### References

1. Dittmann S, Gromyko A, Mikkelsen H, Schaumburg A, Adamian R, Khodakevich L, et al. Epidemic of sexually transmitted diseases in eastern Europe. Geneva: World Health Organization; 1996.
2. Kobyshcha Y. HIV risk-related behavior of homo and bisexual men and STD patients in Ukraine. National AIDS Committee and Center 1994;7:290-3.
3. Normand J, Vlahov D, Moses LE. Preventing HIV transmission: the role of sterile needles and bleach. The effects of needle exchange programs. Washington: National Academy Press; 1995. p. 208-55.
4. Spinhenko Y. Prevention of the spread of AIDS in the Ukrainian SSR. Lik Sprava 1988;9:1-3.
5. Usenko A, Grazhdanov N, Stepanets V, Neshcheret E, Maksiutenko E. Effective knowledge propaganda in the chief strategy for preventing HIV infection among adolescents. Lik Sprava 1994;9:192-6.
6. Tichonova L, Borisenko K, Ward H, Meheus A, Gromyko A, Renton A, et al. Epidemics of syphilis in the Russian Federation: trends, origins, and priorities for control. Lancet 1997;350:210-3.

### Yellow Fever Vaccine

**To the Editor:** Monath et al. (1) outlined existing facilities for distribution of yellow fever vaccines in the United States and pointed to difficulties for prospective vaccinees in remote locations. Their recommendation that primary health-care providers be allowed to dispense yellow fever vaccination merits serious consideration. Acceptance of such a strategy in the United States would inevitably be emulated elsewhere. Nevertheless, before such a strategy is approved, vaccine potency should be monitored at distribution points, and a sample of vaccine recipients should be examined for vaccine-induced immune response.

In Nigeria, systematic investigation of yellow fever vaccine distribution and transportation to remote locations has found loss in vaccine potency. Vaccine in storage sites and immuniza-

